# What Can Local Transfer Entropy Tell Us about Phase-Amplitude Coupling in Electrophysiological Signals?

**DOI:** 10.3390/e22111262

**Published:** 2020-11-06

**Authors:** Ramón Martínez-Cancino, Arnaud Delorme, Johanna Wagner, Kenneth Kreutz-Delgado, Roberto C. Sotero, Scott Makeig

**Affiliations:** 1 Swartz Center for Computational Neurosciences, Institute for Neural Computation, University of California San Diego, La Jolla, CA 92093, USA; adelorme@ucsd.edu (A.D.); joa.wagn@gmail.com (J.W.); smakeig@ucsd.edu (S.M.); 2 Jacobs School of Engineering, University of California San Diego, La Jolla, CA 92093, USA; kreutz@eng.ucsd.edu; 3 Centre de Recherche Cerveau et Cognition (CerCo), Université Paul Sabatier, 31059 Toulouse, France; 4 CNRS, UMR 5549, 31052 Toulouse, France; 5 Computational Neurophysics Lab, University of Calgary, Calgary, AB T2N 4N1, Canada; roberto.soterodiaz@ucalgary.ca

**Keywords:** phase amplitude coupling, cross frequency coupling, information theory, transfer entropy

## Abstract

Modulation of the amplitude of high-frequency cortical field activity locked to changes in the phase of a slower brain rhythm is known as phase-amplitude coupling (PAC). The study of this phenomenon has been gaining traction in neuroscience because of several reports on its appearance in normal and pathological brain processes in humans as well as across different mammalian species. This has led to the suggestion that PAC may be an intrinsic brain process that facilitates brain inter-area communication across different spatiotemporal scales. Several methods have been proposed to measure the PAC process, but few of these enable detailed study of its time course. It appears that no studies have reported details of PAC dynamics including its possible directional delay characteristic. Here, we study and characterize the use of a novel information theoretic measure that may address this limitation: local transfer entropy. We use both simulated and actual intracranial electroencephalographic data. In both cases, we observe initial indications that local transfer entropy can be used to detect the onset and offset of modulation process periods revealed by mutual information estimated phase-amplitude coupling (MIPAC). We review our results in the context of current theories about PAC in brain electrical activity, and discuss technical issues that must be addressed to see local transfer entropy more widely applied to PAC analysis. The current work sets the foundations for further use of local transfer entropy for estimating PAC process dynamics, and extends and complements our previous work on using local mutual information to compute PAC (MIPAC).

## 1. Introduction

A hallmark feature of electrophysiological recordings of brain activity is the presence of rhythmic oscillations [1,2]. Interaction between activity in different frequency bands has been associated with particular brain states and stimulus responses in humans in both healthy and pathological conditions, and more generally in mammalian brains [3]. Until recently, oscillatory dynamics in different frequency bands were, in effect, treated as being largely independent. It is now acknowledged that rhythms at different frequencies can interact temporally both within and between brain structures. More importantly, clearly-defined cross-coupling interactions between neural frequency bands appear across mammalian brain evolution, suggesting they may be supported by a universal evolutionary mechanism serving essential brain functions [1,4]. These facts bring relevance to cross-frequency coupling (CFC) studies, and more importantly, to its most widespread and studied variant, phase-amplitude coupling (PAC).

In phase-amplitude coupling, the phase of a slower rhythm regulates changes in the amplitude of activity at higher frequencies, either within the same signal or between two recorded signals [3]. It is understood that high-frequency oscillations (HFO) emerge from and are topologically constrained within small brain functional areas. In contrast, larger generating areas and/or area-coupled networks are associated with slower rhythms [2]. A consensus view of brain PAC is that activity in disjoint frequency bands interacts by manifesting an architectural hierarchy: low-frequency oscillations manifest or express synchrony within and coherence between large neuronal ensembles, while their phase regulates local changes in faster field activity within brief time/phase windows [1,5]. This pattern of cortical rhythmic interdependence is believed to foster efficient, precisely timed information transmission flow across spatiotemporal scales [4]. Implicit here is also the idea that PAC reflects the causal influence of low-frequency phase on high-frequency amplitude.

PAC has been observed between various frequency bands, in multiple brain regions, under different task conditions and in multiple species (see Table 1 in [3]). In addition to the widespread prevalence of PAC in healthy brain process, links have been found between PAC and a variety of neurological pathologies (e.g., in epilepsy [6], Parkinson’s disease [7,8], Alzheimer’s disease [9], mild cognitive impairment [10], schizophrenia [11,12], and obsessive-compulsive disorder [13]).

Several methods have been proposed to estimate PAC. Although none has been established yet as the gold standard, three methods have been most often used by the scientific community: the Mean Vector Length Modulation Index (MVLmi) [14], the Kullback–Leibler Modulation Index (KLmi) [15], and the General Linear Model Modulation Index (GLMmi) [16]. These methods rely on the assumed covariation of the phase and amplitude time series to statistically estimate PAC presence and strength. One limitation of these approaches is their lack of time resolution. A new PAC estimation method based on mutual information, recently proposed by us in [3]), addresses this challenge. Another limitation of *’correlation’*-based PAC estimation is that it assumes that interactions between the two time series are instantaneous, thus missing the effect of any delayed interplay among frequency bands.

Delays among brain signals arise mainly due to intrinsic information propagation lags through brain circuitry. It has been demonstrated that the brain oscillatory processes are intimately related to these delays, which are believed to constitute an essential mechanism for inter- and intra-brain network synchronization [17]. The delay coordinated activity has proven vital for normal brain function to such an extent that its disruption has been associated with pathologies including multiple sclerosis [18] and schizophrenia [19]. The ability to estimate interaction delays in brain signals may enable estimation of the directionality and causation in the interaction. Thus, the estimation of the dynamics of directed interaction in multi-scale PAC scenarios may help understand PAC’s functional significance.

Concepts from information theory (IT) have proven effective in addressing some current PAC measurement constraints (e.g., [3]). In addition to the advantage provided by the model-free assumptions in the estimation of IT quantities, two specific developments have made information theory especially suitable to addressing some current PAC limitations: (1) Introduction of transfer entropy [20] as a measure of predictive information transfer and interaction delay between time series; and (2) development by Lizier of theory and methods for estimating IT measures in a pointwise or *local* manner [21].

Here, we explore the use of local transfer entropy to study and characterize phase-amplitude coupling dynamics. We aim to provide an initial report of the use of local transfer entropy to study the temporal dynamics of PAC process interactions involving delays. In Section 2, we provide a general background on information theory and introduce the concepts and estimation techniques for transfer entropy as well as its local measure. We then address PAC estimation using transfer entropy (Section 3) and then present simulated and actual data results in Section 4 and Section 5, respectively. In Section 6, we discuss the results and provide general observations on the use and interpretation of TE in the context of PAC.

## 2. Information Theory and Transfer Entropy

**Information theory background.** A central quantity in Information Theory is the Shannon Entropy *H*. To introduce this key concept, let us assume two discrete random variables *X* and *Y* with sets of values *x* and *y*, respectively, probability distributions $p\left(x\right)$, $p\left(y\right)$, conditional probabilities $p\left(x|y\right)$ and $p\left(y|x\right)$, and joint distribution $p\left(x,y\right)$. These quantities are related by p(x,y)=p(x|y)p(y)=p(y|x)p(x). The quantity H(X), which is the average of the “log-surprise” $log1/p\left(x\right)=-log_{2}p\left(x\right)$ for an observation X=x (see Equation (1)), represents the expected uncertainty associated with a measurement *x* of the random variable *X*:

(1) $$H\left(X\right)=-\sum_{x}p\left(x\right)\log_{2}p\left(x\right)$$

Shannon entropy can be extended to two random variables *X* and *Y*; then, the joint entropy can be defined as in Equation (2):

(2) $$H\left(X,Y\right)=-\sum_{x,y}p\left(x,y\right)\log_{2}p\left(x,y\right)$$

It is also convenient to define the notion of conditional entropy as the average uncertainty about *x* that remains when the value of *y* is known (Equation (3)):

(3) $$H\left(X|Y\right)=-\sum_{x,y}p\left(x,y\right)\log_{2}p\left(x|y\right)$$

With these definitions in place, we can then formalize the mutual information (MI) between the random variables *X* and *Y* as a non-negative and symmetric measure defined in Equations (4) and (5):

(4) $$I\left(X,Y\right)=\sum_{x,y}p\left(x,y\right)\log\frac{p(x,y)}{p(x)p(y)}\geq 0$$

(5) $$=H\left(X\right)-H\left(X|Y\right)$$

By assuming a third random variable *Z*—with sets of values *z*, probability distribution $p\left(z\right)$, conditional probabilities $p\left(x|z\right)$ and $p\left(y|z\right)$, and related to *X* and *Y* by p(x,z)=p(x|z)p(z) and p(y,z)=p(y|z)p(z)—one obtains the conditional mutual information (Equation (6)):

(6) $$I\left(X,Y|Z\right)=H\left(X|Z\right)-H\left(X|Y,Z\right)$$

**Transfer entropy.** Now, let us assume the coupled physical system X and Y, whose behaviour is described by the random process *X* and *Y* produces the time series $x_{t}=\left\{x_{1},...,x_{N}\right\}$, $y_{t}=\left\{y_{1},...,y_{N}\right\}$ at the discrete recording times $t\in \left\{1...N\right\}$. With these definitions in place, Wiener’s principle of causality states that, if knowledge about the past of realizations of *X* and *Y* together allows one to predict the future of *Y* better than knowledge about the past of *Y* alone, then a causal influence can be assigned from the process *X* to *Y* [22]. In the information-theoretic framework, this principle can be reformulated as *“What information does the past of X provide about the future of Y that the past of Y did not already provide?”* [23]. The quantity capturing this principle, *transfer entropy*, was formalized by Schreiber [20] in terms of the conditional mutual information (Equation (7)):

(7) $$TE\left(Y\to X\right)=I\left(X^{+};Y^{-}|X^{-}\right)$$

Here, $X^{+}$ is a future random variable of the process *X*, whereas $X^{-}$ and $Y^{-}$ designate the reconstructed past state variables of the process *X* and *Y*, respectively. This quantity as stated in Equation (7) can be viewed as the *average* information contained in the past of the source signal $Y^{-}$ about the next state $X^{+}$ of the destination that was not already contained in *X* past, $X^{-}$. Current extensions of this formulation have been proposed by Wibral et al. [23] and Lizier et al. [24] by assuming: (1) An interaction delay *u* between the time series $x_{t}$ and $y_{t}$; (2) The times series $x_{t}$ and $y_{t}$ can be approximated by a Markov process of order *k* and *l* respectively. With this assumptions, Equation (7) can be rewritten in a more general form [24] as in Equation (8):

(8) $$TE_{Y\to X}^{\left(k,l\right)}\left(t,u\right)=I\left(X_{t}:Y_{t-u}^{\left(l\right)}|X_{t-1}^{\left(k\right)}\right)$$

Transfer entropy is a positive asymmetric quantity whose interpretation is still being debated. However, a consensus seems to be forming around the idea that the quantity provided by TE, far from being interpreted as “true causality”, may be a predictive information transfer [23,25] or predictive causality. These are important concepts that are usually tangled in discussions of information transfers (see [25] for an in-depth discussion). The idea of causal effect may be assumed to mean the extent to which a source process directly drives the next state of a destination process [25]. This can be seen in the action of a falling row of dominoes. On the other side, predictive causality implies one’s ability to predict without committing to a belief in causal efficacy.

**Local transfer entropy.** Most of the information theory quantities currently in use (e.g., entropy, mutual information, transfer entropy) can be seen as an spatial or time average of more fundamental *local* or *pointwise* information bearing quantities. Local information theoretic measures characterize quantities from a specific, localized subset of measurements *x* and *y* of the random variables *X* and *Y*, rather than the associated average measure computed over all available data [26]. For example, local mutual information values $i\left(x,y\right)$ (Equation 9) may be averaged to compute overall MI $I\left(X,Y\right)$ (Equation (10)):

(9) $$i\left(x,y\right)=\log_{2}\frac{p\left(x|y\right)}{p\left(x\right)}$$

(10) $$I\left(X,Y\right)=E_{X,Y}\left[i\left(x,y\right)\right]$$

Since the TE is just a conditional MI (see Equation (7)), local transfer entropy can be defined as the pointwise conditional mutual information computed from an specific source state $y_{t-u}^{\left(l\right)}$ to a specific target event $x_{t}$ conditioned by the event state history of the target $x_{t-1}^{\left(k\right)}$ [24] as in Equation (11):

(11) $$te_{Y\to X}^{\left(k,l\right)}\left(t,u\right)=i\left(x_{t}:y_{t-u}^{\left(l\right)}|x_{t-1}^{\left(k\right)}\right)$$

While the TE is a strictly positive quantity, the local transfer entropy may be either positive or negative, indicating whether or not the source $y_{t-u}^{\left(l\right)}$ is providing informative information regarding the set $x_{t},y_{t-u}^{\left(l\right)},x_{t-1}^{\left(k\right)}$. When TE is negative, Lizier [27] pointed out that the source element is actually *misleading* about the state transition of the destination. Other instances where other causal information sources influence the destination, or in stochastic systems, may lead to negative TE values. Given this, we may not lose any clear insight into the interaction of our variables of interest by neglecting negative TE values, as we will do here.

One of the practical advantages of the local transfer entropy is that it provides information about the dynamics of the information transfer while at the same time allowing recovery of the interaction delay δ between the time series of the system analyzed as in Equation (12) [26]:

(12) $$\delta =\underset{u}{argmax}\left(TE_{Y\to X}^{\left(k,l\right)}\left(t,u\right)\right)$$

These features will be exploited in this manuscript to analyze the dynamics of the PAC process.

**Estimating transfer entropy**. Estimation of transfer entropy is usually carried out through the use of mutual information and conditional mutual information estimation methods. The simplest and most widespread estimators for MI are extensions of algorithms to compute entropy based on straightforward plug-in evaluation of defining densities by its empirical estimates (called the *plug-in* estimator by [28]). Another popular branch of entropy estimation methods use a similar principle but estimate the underlying densities by (1) kernel estimation methods (*KDE*) [29,30] or (2) by taking advantage of the geometry of the space jointly formed by the support of the variables used in the computation, approximating the densities at the point *x* using the volume defined by a sphere encapsulating its *K* nearest neighbors (known as K-nearest neighbors, *K-NN* [31,32,33]). Despite its widespread popularity, this family of methods is known to have serious bias problems [34,35,36,37,38]. Recent advances in the development of MI nearest-neighbour estimators, specifically that proposed by Kraskov, Stogbauer, and Grassberger (KSG) [33] have provided an alternative to substantially improve the problem of the bias by providing a method that effectively bypasses the need to estimate densities. The KSG estimator builds on the nearest-neighbors-based Kozachenko and Leonenko entropy estimator (KL) [38], which Kraskov et al. modified to make the bias resulting from the nonuniformity of the densities in marginal spaces cancel each other. To do this, Kraskov et al. observed that, for any fixed K value, the distance to the Kth neighbor in the joint space is larger than the distances to the neighbors in the marginal spaces, which lead to use different distances scales in the joint and marginal spaces when using the KL estimator for computing MI [33]. Consequently, Kraskov et al. recommended not to use a fixed value of K for the marginal entropy estimation and proposed a K-NN MI estimator in (Equation (13)):

(13) $$I\left(X,Y\right)=\psi \left(K\right)-\langle \psi \left(n_{x}+1\right)+\psi \left(n_{y}+1\right)\rangle +\psi \left(N\right)$$

Here, *N* is the number of samples of *X* and *Y*, and ψ denotes the digamma function ($\psi \left(x\right)=\Gamma {\left(x\right)}^{-1}\frac{d\Gamma \left(x\right)}{dx}$). The terms $n_{x}\left(i\right)$ and $n_{y}\left(i\right)$ designate the number of samples falling into a strip of the marginal space of *X* and *Y*, respectively, defined by the distance to its *K* nearest neighbors. MI values $I\left(X,Y\right)$ are returned in nats. For a detailed derivation of the method, see [33] or [3].

The KSG estimator constitutes an effective non-parametric estimator of MI that is data efficient (resolving structures down to the smallest possible scales), adapts resolution (binning scale changes according to the underlying data point density), and has minimal bias [33]—is indeed unbiased for independent variables. The neat formulation of the KSG estimator allowed Lizier [26] to extend it to compute local mutual information by unrolling the expectation (〈...〉) in Equation (13), yielding Equation (14):

(14) $$i\left(x,y\right)=\psi \left(K\right)-\psi \left(n_{x}+1\right)-\psi \left(n_{y}+1\right)+\psi \left(N\right)$$

An extension of this formulation has been proposed for the computation of local transfer entropy through the direct estimation of conditional mutual information in the form of Equation (15):

(15) $$i\left(x,y|z\right)=\psi \left(K\right)-\psi \left(n_{xz}\right)+\psi \left(n_{yz}\right)-\psi \left(n_{z}\right)$$

In this manuscript, we used this formulation proposed by Lizer for the computation of TE [27] as implemented in the JIDT Toolbox [39].

**Active information storage.** From a corollary of Wiener’s causality principle in the IT context, it can be derived that, for the values of TE to be interpretable in the context of Wiener causality, it is necessary to ensure that a signal can optimally predict its own future behavior. It is then convenient to introduce an IT quantity to describe how well a signal can predict itself. This is the aim of the active information storage (AIS) introduced by Lizier [21], which defined a measure of how much of the information from the past of the process *X* is observed to be in use in determining its *next observation*. Formally, the AIS can be defined as the expected mutual information between realizations $x_{n}^{\left(k\right)}$ of the past state $X_{n}^{\left(k\right)}$ and the corresponding realizations $x_{n+1}$ of the process *X* (Equation (16))

(16) $$AIS_{x}\left(k\right)=I\left(X_{n}^{\left(k\right)}:X_{n+1}\right)$$

Given a range of values for the state’s length parameters *k* and *l*, we use the maximal values of AIS to estimate the optimal value of the state’s lengths that ensures optimal signal self-prediction.

## 3. Approaching PAC Estimation with Information Theory Local Measures

We now study and characterize the temporal dynamics of directed information transfer (from phase to amplitude) in the PAC process through local transfer entropy. Note that we assume only directed phase to amplitude interactions. This choice is discussed in Section 6.3.

Most methods for computing PAC follow a similar data processing pipeline. First, high and low-frequency band signals are extracted for the two frequency ranges in which the PAC coupling is to be assessed. These frequency bands are centered on a lower central frequency $f_{phase}$ for the phase time series, and a higher central frequency $f_{amp}$ for the amplitude time series. For this, either band-pass filtering or time-frequency decomposition can be used with similar results. Then, instantaneous phase and amplitude time series are obtained from the low- and high-frequency band signals, respectively, using the Hilbert transform or else directly from the complex signal when time-frequency decomposition has been used. These time series are then used to compute a PAC measure. After the PAC measure is computed, a statistical analysis is usually carried out by comparing the estimated PAC value with a distribution of surrogate PAC values calculated under a no-PAC assumption. Surrogate values are obtained by computing PAC from the original input signals after shuffling the two time-series many times as to destroy any PAC relationship [15]. Significance is then assessed by determining whether the estimated PAC measure for the actual data belongs or not to the distribution of surrogate PAC values. Here, we will follow this same preprocessing pipeline.

We have recently proposed and validated a method, MIPAC, to estimate time-resolved PAC using local mutual information [3]. Estimating PAC through MIPAC begins by computing two time series capturing instantaneous phase and amplitude in the two frequency bands of interest. Then, local mutual information is computed between these two signals following Equation (14). When computing Equation (14) in MIPAC, rather than the Euclidean norm used for the instantaneous amplitude, a circular norm [40]—to account for its periodic nature– is used for the phase component to compute and find the nearest neighbors in the marginal space defined by the support of the instantaneous phase. Finally, the local mutual information time series is low-pass filtered under $f_{phase}$.

Our analysis using local transfer entropy resembles that used for MIPAC [3]. Again, it begins by computing two time series capturing the instantaneous low-frequency phase and high-frequency band amplitude. To assess phase-to-amplitude information transfer and estimate the delay in the interaction between these frequency features, we first estimate the TE for a range of delays *u*. Given the periodic nature of the frequency components in question, a plausible range of delays may be defined by a time range smaller than the span of a full cycle of the lower (phase) frequency. Since the TE is maximal when the parameter *u* is the actual interaction delay δ [23], we choose to analyze the local transfer entropy time series corresponding to the *u* that maximizes the TE. Statistical significance is then computed at each latency as described previously.

Before TE computation, special consideration should be given to selecting the state history length for each signal. Although a few algorithms and methods have been proposed to estimate these lengths (e.g., [41,42]), a consensus on choice of method is far from being reached [26]. In our work, we estimate the history lengths (also called ’*embedding parameters*’) *k* and *l* by determining empirically the embedding values that maximize the AIS in the instantaneous phase and amplitude. Since the KSG algorithm is bias corrected by construction, the values of *k* and *l* that maximize the TE should successfully capture the corresponding past states’ relevant information. The estimated history values, along with an estimate of the nearest neighbors parameter *K*, are used then to estimate the delay *u* as described previously. In the current manuscript, data processing, simulations, computation, and analysis were performed using EEGLAB [43], functions from the PACTools plug-in to EEGLAB [44], and custom scripts written in MATLAB (The Mathworks, Inc.). The JIDT Toolbox [39] was used to compute local TE and AIS.

## 4. Simulation Results

**Simulating PAC.** Simulated PAC signal coupling was generated by following [45]. Here, coupling in the signal was simulated between amplitude frequency $f_{Amp}=70$ Hz and phase frequency $f_{phase}\;=6$ Hz with a sampling frequency of fs=1000 Hz. The lower frequency component, or modulator $S_{\phi }$ was built by band-pass filtering a Gaussian white noise signal around $f_{phase}$ assuming a $\Delta f_{phase}=1$ Hz bandwidth. The filter consisted in a Hamming-windowed (sinc) FIR notch filter implemented in the EEGLAB function *pop_eegfiltnew.m* [43]. The modulator signal was normalized to have unit standard deviation $\sigma_{phase}=1$ before computing the cosine of its instantaneous phase obtained by using the Hilbert transform. The resultant modulator signal $S_{\phi }$ was then used to generate the high frequency component, or carrier $S_{A}$. For this, a sinusoid with frequency $f_{Amp}=70$ Hz was modulated by using a sigmoid fed by $S_{\phi }$ as in Equation (17):

(17) $$S_{A}\left(t\right)=\frac{1}{1+exp\left(-\lambda S_{\phi }\left(t\right)\right)}$$

Here, the parameter λ=6. We then introduced a delay between $S_{A}$ and $S_{\phi }$ by shifting $S_{A}$τ ms forward respect to $S_{\phi }$. In the remainder of the text, τ=30 ms if not otherwise specified. Finally, to obtain the simulated PAC signal (N = 5000 samples), we added both the delayed $S_{A}$, $S_{\phi }$ and a Gaussian white noise with power of 0.5 dBW, as implemented in MATLAB function *wgn*, to yield a resultant signal with SNR=23.7.

**Figure 1:**
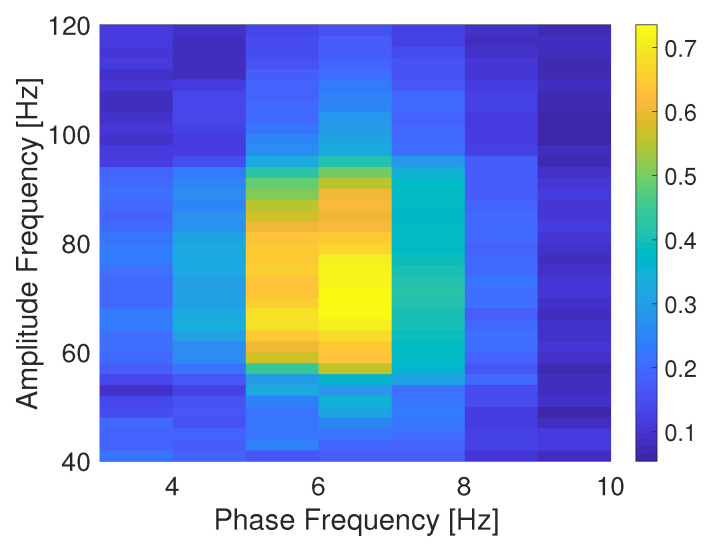
Comodulogram for the simulated PAC signal. Comodulogram depicting the time-averaged mutual information based phase amplitude coupling (MIPAC) computed between phase frequencies from 3 to 10 Hz (1-Hz steps) and amplitude frequencies from 40 to 120 Hz (10-Hz steps) for the simulated signal.

**Simulation results**. A continuous PAC simulated signal was generated as indicated at the beginning of this section. Figure 1 shows the time-averaged mutual information phase amplitude coupling (MIPAC) computed between phase frequencies from 3 to 10 Hz (1-Hz steps) and amplitude frequencies from 40 to 120 Hz (in 10-Hz steps) in the simulated signal. The figure shows that the coupling has been effectively introduced between amplitude frequency $f_{Amp}=70$ Hz and phase frequency $f_{phase}=6$.

**Figure 2:**
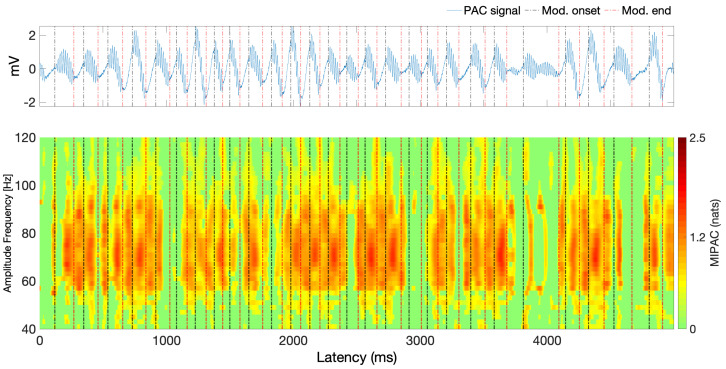
Simulated PAC signal and MIPAC. *Top panel* shows the simulated PAC signal with coupling between amplitude frequency $f_{Amp}=70$ Hz and phase frequency $f_{phase}=6$ Hz using a sampling frequency of fs=1000 Hz. Here, the phase component is delayed 30 ms with respect to the amplitude component. Vertical black and red dotted lines denote the onset and end of HFO bursts. *Lower panel* shows the MIPAC computed between $f_{phase}=6$ Hz and amplitude frequencies from 40 to 120 Hz (10-Hz steps). Despite the delay between the frequency components, MIPAC seems able to detect the temporal evolution of PAC in the simulated signal. MIPAC appears to peak roughly by the end of the HFO events with some instances were the peaks persist after the culmination of the HFO. In these cases, a *reset* of the MIPAC appears to occur with the onset of the following HFO. All non-zero values are statistically significant (p<0.05, uncorrected) as per a test performed using 100 surrogates values. Non-significant values were set to zero.

In Figure 2, we show the simulated PAC signal (top panel) and the time course of the MIPAC computed for $f_{phase}=6$ and amplitude frequencies from 40 to 120 Hz (10-Hz steps) (lower panel). Black and red lines here depict the beginning and end of the high frequency oscillation (HFO) bursts. In this case, we see that from the perspective of the MIPAC, PAC seems to be present along the whole signal while missing some HFO around 1000 ms and 4000 ms. Roughly, the MIPAC seems to peak after HFO onset, but this is not strict, since MIPAC peaks appear following the HFO in some instances. In these instances, MIPAC appears to be *reset* by the onset of the following HFO.

**Figure 3:**
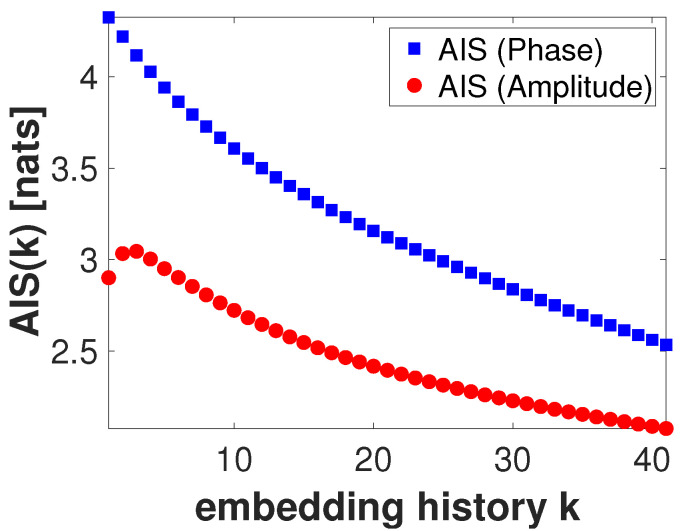
Active information storage as a function of the embedding history *k*. Active information storage (AIS) computed for the instantaneous phase at $f_{phase}=6$ Hz (blue squares) and instantaneous amplitude $f_{Amp}=70$ (red dots). Peaks in the AIS suggest that an embedding history of k=1 for the phase and l=3 for the amplitude are adequate to capture the relevant past history. All values shown are statistically significant (p<0.05, uncorrected) as per a test performed using 100 surrogate values.

To characterize the self-prediction ability of the instantaneous phase and amplitude components, at $f_{phase}=6$ Hz and $f_{Amp}=70$ Hz respectively, for a given value of nearest neighbors K=50, we compute the active information storage as a function of the history length parameter *k*. Unless otherwise specified, throughout the manuscript, significance testing was carried out by generating 100 surrogate signals whose duration and number of samples match those of the signal for which statistical significance is being assessed. The results of this computation are shown in Figure 3.

**Figure 4:**
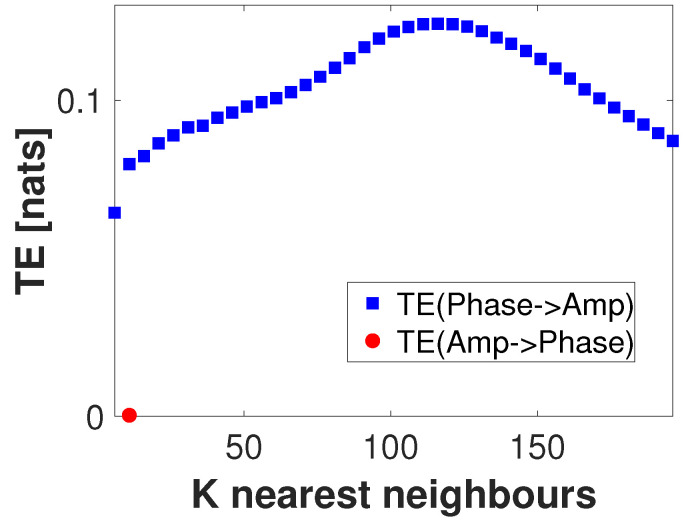
Transfer entropy as a function of K-nearest neighbors. Transfer entropy computed from the low frequency phase component at $f_{phase}=6$ to the high frequency amplitude component at $f_{Amp}=70$ (blue square), and in the opposite direction (red dots), as a function of the *K*-nearest neighbors values. Embedding history of k=1 for the phase and l=3 for the amplitude were used. Only significantly positive values are shown (p<0.05, uncorrected). Information transfer from phase to amplitude appears to be predominant, independent of the value of *K*.

In Figure 3, all computed AIS values for both instantaneous phase and amplitude were statistically significant (p<0.05, uncorrected) as per a test performed using 100 surrogate values. The maximum AIS values for instantaneous phase and amplitude appear to suggest that k=1 for phase, and k=3 for amplitude ensure an optimal self signal prediction. In the following, we will use these peaks on the AIS as values for the history length parameters for instantaneous phase (k=1) and amplitude (l=3). After testing with multiple values for nearest neighbors (not shown here), this result appears to be stable with respect to the parameter *K*. Next, for the given embedding parameters (k=1 and l=3) and delay u=30 ms, we compute the TE in both directions between instantaneous phase and amplitude for a number of values of *K* (nearest neighbors). Significance testing was carried out by generating 100 surrogate values. Figure 4 shows the results of this computation. Here, only positive and significant (p<0.05, uncorrected) TE values are shown. Note that, independent of the value of *K* used, transfer of information from phase to amplitude appears to be dominant, peaking at K=116. In the remainder of this analysis, the transfer of information from amplitude to phase will not be considered.

**Figure 5:**
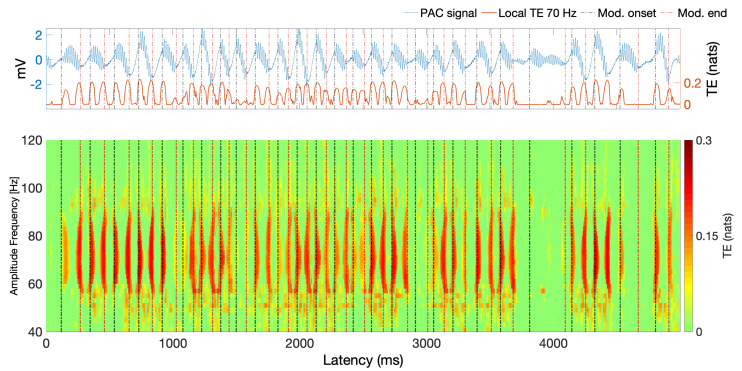
Simulated PAC signal and local TE. The *top panel* is similar to the top panel in Figure 2 and shows the original simulated signal in blue, the black and red vertical dotted lines marking HFO burst onsets and offsets. *Bottom panel* shows the local TE computed from the simulated signal’s instantaneous low frequency phase at $f_{phase}=6$ to its instantaneous amplitude in the 40–120 Hz band. Significant TE values here appear at the beginning and end of HFO events. For reader’s convenience, we have added to the upper panel (see red solid trace) the time course of the computed local TE at 70 Hz. All non-zero values are statistically significant (p<0.05, uncorrected) as per a test performed using 100 surrogate values. Before plotting, non-significant values were set to zero.

The lower panel of Figure 5 shows local TE computed from instantaneous phase at 6 Hz to amplitude in a band from 40 Hz to 120 Hz. The upper panel is similar to that in Figure 2, showing the simulated signal and the onset and offset of the HFO (red and black vertical dotted lines). For the TE computation, we used the parameters estimated in the previous analysis: k=1, l=3, u=30 and K=116. We can see that, despite similarities to the MIPAC results in Figure 2, the local TE seems to peak at HFO burst onsets and offsets. Similar to the behaviour of MIPAC in Figure 2, the local TE measure misses the HFO events occurring near 1000 and 4000 ms.

**Figure 6:**
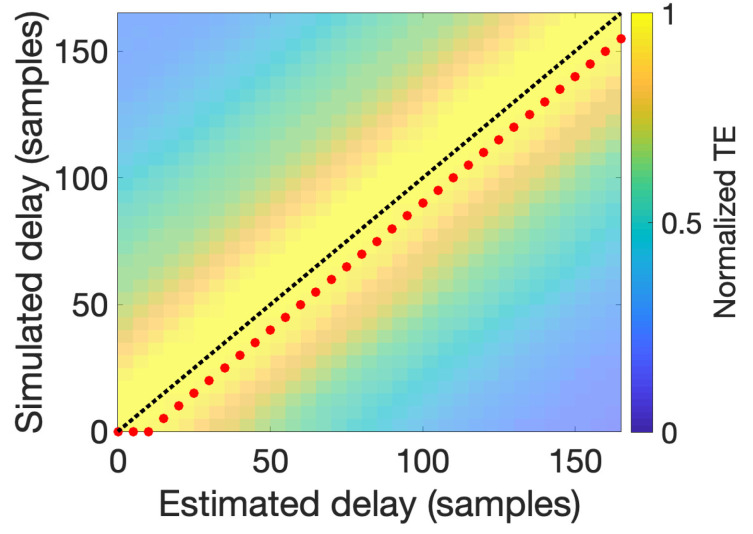
Delay estimation. Transfer entropy estimated using different delays ranging from 0 to the latency (166 time points) corresponding to one full cycle of the phase frequency component (here at $f_{phase}=6$ Hz) for a set of simulated signals generated using the same delay values (*y*-axis). For each simulated signal, the maximum estimated TE value is indicated by a red dot; this yielded the best estimated delay. Estimated values appear to be consistently close to optimal estimation performance, indicated by the black diagonal dotted line.

Finally, to test the capability of TE to recover different delays, we simulated a PAC signal with the same parameters as described at the beginning of the current section but with different delays *u* between the phase and amplitude components. These delays ranged from 0 to a delay corresponding to one full cycle of the phase frequency component at $f_{phase}=6$ Hz (in this case, 166 time points). Delay estimation followed as described at the end of the Section 3, by using a range of delays ranging from 0 to 166 points and the parameters *k*, *l*, and *K* used previously. Figure 6 shows normalized TE values using a color code, as a function of the delay values used in the simulation (*y*-axis) and subsequent estimation (*x*-axis). The red dots denote the maximum TE achieved for each simulated signal given the estimated delay, and indicate the best estimated delay. Ideally, estimated and simulated delays would meet at the black diagonal dotted line. From our results, the procedure followed appears to successfully recover the simulated delay, having a maximum deviation from the real simulated delay of only ten samples, equivalent to 10 ms (see Section 6.1 for further discussion).

## 5. Estimating PAC with Transfer Entropy from Actual ECoG Data

To evaluate our findings in real data, we applied the same methodology as in Section 4 to actual electrocorticographic (ECoG) data from a human subject.

**Figure 7:**
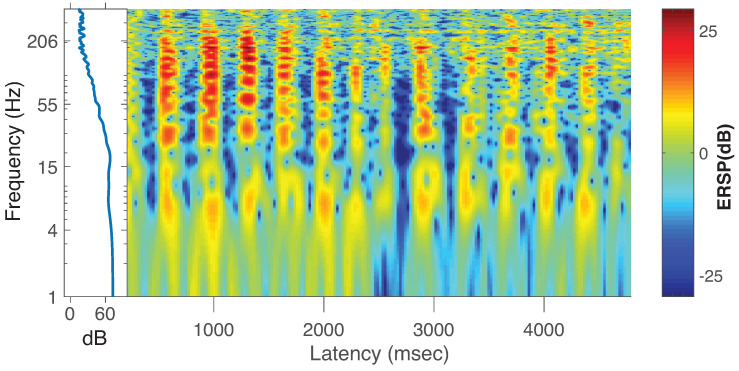
Time-frequency features of the data clip. Spectral characteristics of the 5 s data clip obtained through a wavelet decomposition implemented in EEGLAB function *newtimef.m*.

**Data collection**. Electroencephalographic (EEG) recording from a single subject undergoing pre-surgical epilepsy evaluation at the North Shore University Hospital, Long Island Jewish Medical Center (NY) was performed using intracranial electrodes at a sampling rate of 1999 samples per second per channel. Data were referenced to common-average reference. Seizure detection algorithms were used; the data were also reviewed by an EEG technician and a physician. From a labeled data clip recorded during an epileptic seizure, 5 s of data (*n* = 9995 samples) were extracted from a recording at an electrode (label: Tm2) located in the temporo-medial area (label: Tm2). Figure 7 shows the spectral characteristics of the data clip in an event-related spectral perturbation (ERSP) plot [46] obtained through a wavelet decomposition as implemented in EEGLAB function *newtimef.m*. We can see a rhythmic low frequency activation at 4–8 Hz simultaneously with a broadband (30–250 Hz) activation from this figure. We will refer to these frequency bands as low and high, respectively.

**Figure 8:**
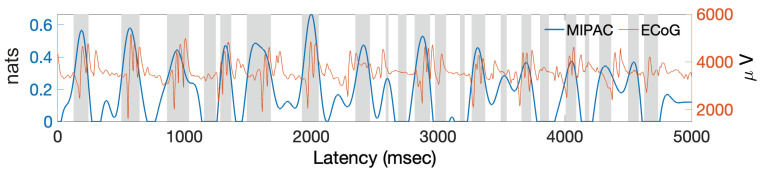
Time course of ECoG data and MIPAC. The red trace shows the 5 s time course for one ECoG data channel during an epileptic seizure. The blue trace shows MIPAC computed between low-frequency ECoG signal phase (at 4–8 Hz) and high frequency amplitude (30–250 Hz). Statistically significant MIPAC values (p<0.05, uncorrected, 100 surrogates) are shown in light gray.

**Computing MIPAC**. Figure 8 shows MIPAC computed between the low (4–8 Hz) and high (30–250 Hz) frequency bands of the data clip shown in Figure 7. Instantaneous phase and amplitude were extracted from the low and high frequency ranges respectively as detailed in Section 3. Here, the time courses of the ECoG signal and of MIPAC are shown in red and blue, respectively. Significance computed using 100 surrogates (p<0.05, uncorrected) appears in light gray. As we expected, increases in MIPAC correspond to HFO bursts appearing at a similar phase of the low frequency oscillation.

**Figure 9:**
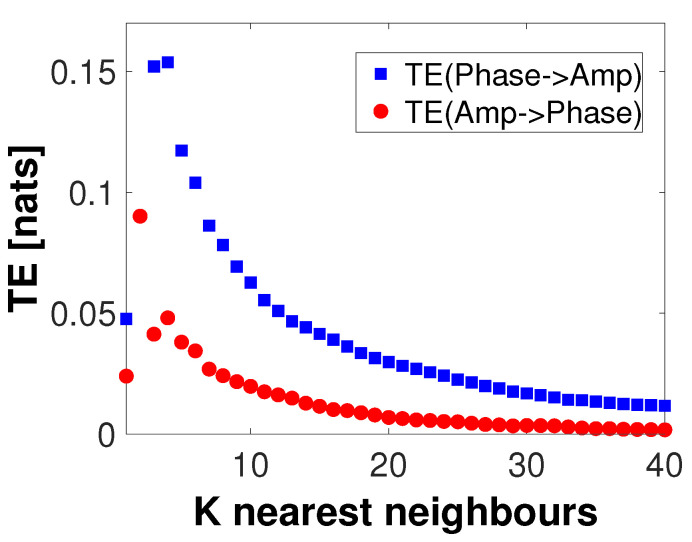
Transfer entropy in ECoG data as a function of K-nearest neighbor values. Transfer entropy computed on the 5 s data clip for a range of K-nearest neighbor values between instantaneous phase at 4–8 Hz and instantaneous amplitude in the range 30–250 Hz. Information transfer from phase to amplitude is designated with blue squares, while red circles designate transfer in the opposite direction. The process seems dominated by flow of information from phase to amplitude, although there is some flow of information in the opposite direction. Each value shown is statistically significant (p<0.05, uncorrected) as per a test performed using 100 surrogates.

**Estimating PAC using local TE.** Using the same instantaneous phase and amplitude derived from the low- and high-frequency bands defined previously, analysis was carried out as in Figure 3 and Figure 5. First, AIS was computed for embedding history lengths from 1 to 50 (not shown). Retaining the embedding history parameters corresponding to the highest AIS, their values were set to k=3 (phase) and l=3 (amplitude). Next, we computed transfer entropy in both directions, from phase to amplitude and from amplitude to phase, as a function of the *K* nearest neighbor parameter in the range K=1 to 40 (Figure 9). The resulting information transfer was predominantly from phase to amplitude, independent of the value of *K*. However, some information transfer from amplitude to phase was also found.

**Figure 10:**
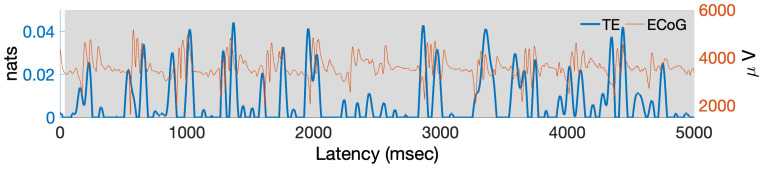
Time courses of the ECoG data and of local TE between low frequency phase and high frequency amplitude time series. The red trace shows the time course of the 5 s ECoG data clip recorded during an epileptic seizure. Computed local transfer entropy (from phase to amplitude) is shown in blue. The local TE time course seems to peak at onsets and offsets of HFO bursts in the ECoG signal. Statistically signficant TE values (p<0.05, uncorrected, 100 surrogates) are shown in light gray.

Next, we computed the TE from instantaneous phase to amplitude using the parameters *k* and *l* from the AIS analysis and K=100, for a range of delay values ranging from 0 to 195 samples (corresponding to half a cycle of the central frequency, $f_{phase}=6$ Hz, of the lower frequency band). The maximum TE in the delay range studied corresponded to a value of u=85 samples (not shown). We selected this delay value along with the values of *k*, *l* and *K* previously used to compute the local transfer entropy from instantaneous phase to amplitude (Figure 10).

In Figure 10, the time course of the signal and the local TE are shown in red and blue, respectively. Significance testing (p<0.05, uncorrected) for local TE, carried out using 100 TE surrogates, is shown in light gray. Local TE values were filtered below 12 Hz (roughly two times the central frequency of the lower frequency band) for better visualization of their main features. As can be seen here, local TE increases and peaks roughly at the beginning and end of HFO bursts. This result resembles that obtained for the simulated signal in Figure 5. An apparent decline of TE values between 2000 ms and 3000 ms appears inconsistent with the MIPAC results shown in Figure 8.

## 6. Discussion

### 6.1. Computing TE and MIPAC in Simulated PAC Data

In Section 4, we simulated a PAC signal (sampling rate, 1000 Hz) in which the instantaneous phase at $f_{phase}=6$ Hz modulated the instantaneous amplitude at $f_{amp}=70$ Hz. A delay of u=30 samples (equivalent to 30 ms) was introduced to simulate a causal interaction from phase to amplitude. Using this signal, we computed mutual information-based phase-amplitude coupling (MIPAC) for all combinations of phase frequencies from 3 to 10 Hz (in 1-Hz steps) and amplitude frequencies from 40 to 120 Hz (in 10-Hz steps). A comodulogram conformed with the temporal average of MIPAC values (Figure 1) confirmed the existence of PAC at the selected frequencies. Comparing MIPAC time series to the time course of the simulated signal for $f_{phase}=6$ Hz and $f_{amp}=70$ Hz, we found that increases in MIPAC corresponded to bursts of simulated (70 Hz) HFO. Next, we computed bidirectional transfer entropy between phase ($f_{phase}=6$ Hz) and amplitude ($f_{amp}=70$ Hz). A critical step when computing transfer entropy is determining the signal’s history length, or embedding parameters. Several methods have been proposed to estimate these values, but none has produced a consensus as to the best estimation method. Here, we explored the space of parameters that maximize signal *self*-prediction, as measured by active information storage (AIS) [21], to obtain the embedding parameters we used for instantaneous phase (*k*) and amplitude (*l*) (see Figure 3). To compute the AIS, we assumed a value for the nearest neighbors of K=50. However, the value of *K* seems not to be relevant, as AIS is very robust to the selection of this parameter [47]. Using the estimated parameters *k* and *l*, we computed transfer entropy as a function of the nearest-neighbor parameter *K* in both directions, from phase to amplitude and from amplitude to phase (Figure 4). We found that, in the simulated signal, the transfer of information from phase to amplitude was dominant, independent of the selected value of *K*. TE’s dependency on the parameter *K* seemed to be described by a concave function with a maximum at K=116, which we used in further computations.

At this point, it is important to recall that TE estimation was carried out using the KSG estimator implemented in the JIDT toolbox [39], and, when using this estimator, TE is defined to be the average value of the local TE. In this case, we found that the *K* value corresponding to peak TE in Figure 4 was suitable for describing the process simulated, but using lower *K* values to estimate local TE led to increased variance in the time series. This fact brings to the table the issue that how to select the number of neighbors in the KSG algorithm is still under discussion.

Using parameters *k*, *l*, and *K* estimated in the previous analysis, we replicated the analysis in Figure 2 using local TE. Since in this case the flow of information *from phase to amplitude* was dominant (Figure 5), we only computed local TE in this direction. We found that, for a range of frequencies near $f_{amp}=70$ Hz, phase at $f_{phase}=6$ Hz is related to increased local TE near onsets and ends of HFO signal bursts. This result, as we can see, differs from the time course of MIPAC, which seems to most often reach its maximum local value during HFO bursts.

As it was demonstrated by Wibral et al. [23], TE is maximal when the delay parameter *u* is equal to the true interaction delay δ [23]. This principle can be used to estimate the interaction delay between the process involved in the TE computation by exploring the space of the parameter *u*. In Figure 6, we simulated several signals using the same parameters as in Figure 2 but varying the delay between instantaneous phase and amplitude in a range from zero to 116 samples, corresponding to a full cycle of the phase frequency. For each of these signals, TE was computed for a range of *u* varying from zero to 116 samples. The TE maximum was indicated by red dots in Figure 6, while the true delay was indicated by the diagonal dashed black line. For each signal, we obtained a plateau of maximum TE values around the true simulated delay value, with the maximum deviation in the value of *u* being no bigger than 10 ms. It is obvious from Figure 6 that the maximum TE values always underestimate the real value and that an almost constant bias of 10 ms seems to be present. The cause of this bias is not clear and will be the subject of planned future study. However, we note that the 10-ms bias or deviation is on the order of the generation of action potentials [48].

### 6.2. Estimating PAC with Local TE in Actual ECoG Data

To evaluate the use of local TE in estimating PAC in actual ECoG data, we used a brief recording from intracortical electrodes (ECoG) in a single patient undergoing pre-surgical monitoring for epilepsy (see Section 5). We used five seconds of data during a clinician-labeled seizure from a single electrode placed in the medial temporal brain region and referred to average reference across the ECoG recording array. The spectral characteristics of the signal in Figure 7) featured two main frequency ranges having significant activity, the low-frequency 4–8 Hz theta band and across a high-frequency broadband range (30–250 Hz) [49]. These two frequency ranges were used to first extract instantaneous (low-frequency) phase and (high-frequency) amplitude, and then to compute PAC using the MIPAC method (Figure 8). As in the simulated signal, statistically significant increases in MIPAC corresponded clearly to bursts of high-frequency activity in the signal. This result was later compared to the local TE-derived PAC estimate.

To estimate local TE between these frequency ranges, we carried out an analysis similar to what we performed (Figure 3) to estimate the embedding parameters for phase (*k*) and amplitude (*l*), which suggested using k=l=3. These values were used to study the relationship of bidirectional TE to the nearest neighbor parameter *K* (Figure 9). Contrasting with the simulated signal results (Figure 4), for the actual signal, we found statistically significant information transfer between phase and amplitude in both directions, though that from phase to amplitude dominated. This result was preserved over the range of *K* studied (up to K=100). Next, we investigated the local TE for K=2, where the TE between phase and amplitude in both directions was maximal, and confirmed that the variance of the local TE was considerably higher than for higher *K* values (not shown), and that TE features appeared to become independent of *K* for values above K=40. Based on this, we decided to use the value K=40 as well as k=l=3 in our next computation. Then, we computed local TE from phase to amplitude using the same frequency components (Figure 10). The result replicated our finding for the simulated signal in which the local TE peaks occurred at high-frequency burst onsets and offsets. We speculate that these information transfer maxima at the beginnings and ends of high-frequency bursts reflect the causal role of the low-frequency oscillation controlling the appearance of the bursts, most likely indicating that firing of neurons in the local neighborhood is thereby constrained within a small time window (a few 10 s of ms) at times at which the relatively large theta frequency swings produced a biasing potential that encouraged neural spiking across neurons in the affected cortical domain. Thereby, spike impulses from the PAC-exhibiting area, upon reaching common target neurons in near synchrony, should be more effective in affecting the activity of the target neurons, possibly enhancing short and long-term plasticity in those neurons and shaping effective coupling strength of these network pathways [50,51,52].

### 6.3. The Problem of Directional Causality in PAC

In our work, we have only assumed the flow of information directed from low-frequency phase to high-frequency broadband amplitude, disregarding the potential opposite interaction suggested recently by [53]. This choice is in line with an overwhelming amount of experimental work published in the last years indicating and providing models to support phase-to-amplitude coupling (e.g., [52,54,55,56,57]). For example, neurophysiological evidence for the modulation of high-frequency amplitude by the phase of slow neuronal oscillations has been observed in the interaction between slow neocortical oscillations and thalamocortical rhythmic burst firing (spindles) during slow-wave sleep [54]. Slow oscillations are composed of synchronized states alternating between hyperpolarization (down-state) and depolarization (up-state) and spread through the neocortex travelling to the thalamus, where they promote a pattern of increase and decrease in rhythmic burst firing. Thus, the spindles’ temporal evolution is continuously initiated, shaped, and terminated by slow-wave-promoted corticothalamic feedback (for reviews, see [52,54,55]), providing evidence for the modulation of high frequency amplitude through oscillatory phase.

Another example supporting phase-to-amplitude interaction comes from work of Schroeder and Lakatos, [56,57] examining coupling between gamma band (30–100 Hz) amplitude and phase at delta (<3 Hz) or theta (4–7 Hz) frequencies [2,14]. Slow oscillations in the cortex can become entrained to external rhythms, thus aligning high excitability phases or up-states to occurring or expected external events so as to enhance their sensory processing. During the slow-oscillation high-excitability phase, gamma band (or high-frequency broadband) amplitudes may be enhanced such that a gamma burst occurs at the time when a task-relevant input is expected. Since gamma-band activity appears to be more metabolically-demanding than low-frequency oscillations [58,59], the coupling between gamma and the slower phase is believed to ensure that information transfer resources are used efficiently and high-frequency activity (and concomitantly, spike signaling using local neuronal resources) is selectively enhanced at critical time points. This suggests that slow oscillations act as gatekeepers for local high-frequency (and spiking) activity, thus suggesting a phase-to-amplitude causal relationship (for a review see [60]).

It is appropriate to recall that PAC has also been demonstrated in nonlinear oscillators [61] in which there is no specific modulation process or known mechanism for generating such an effect, the nonlinear dynamics of the system itself thus being the most probable cause of the PAC phenomenon. Therefore, PAC results (including those obtained through information theory-based methods) must be interpreted cautiously, especially if there is no physiologically plausible mechanism or model explaining the process. We acknowledge that a causal relationship from high-frequency amplitude to low-frequency phase might exist, but we argue that this should come from some common driving activity operating with different delays in the high- and low-frequency ranges in which PAC is assessed.

### 6.4. MI and TE, Two Faces of the PAC Process?

In both our simulated and actual data analyses, MIPAC indicated a continuous modulation process with a local maximum within the time window of the modulated high-frequency bursts. On the other hand, TE peaks appeared at the beginnings and ends of these periods, though in some instances (see Figure 10, 2000–3000 ms), where MIPAC indicated a coupling, TE failed to display this behavior. These results pose the question of what information about PAC in cortex are provided by instantaneous MIPAC (a form of local mutual information) and by delay-estimating transfer-entropy based phase-amplitude coupling (TEPAC). Recall that local mutual information captures both linear and nonlinear statistical relationships between the two time series involved in its computation, while positive transfer entropy indicates a *weak* causality or predictive information transfer between them. These two principles are thus not in contradiction, nor are our PAC results. We hypothesize that the flow of information at the beginning of the modulation process, as measured by local TE, initiates or facilitates the process that MIPAC measures. However, the mechanisms responsible for this facilitation remains unclear, and this idea may conflict with the implicit assumption that the PAC phenomenon is a continuous process carried out and determined by the influence of the timing of neural oscillations on a brain area’s cell ensemble. Another option is that TE may not represent a physical process in the PAC phenomena, and the results obtained are just a statistical characterization of the modulation process without further information on the actual biophysical interactions. In the current manuscript, we do not address these questions but rather here provide an initial report characterizing PAC computed using local transfer entropy. We do believe, despite these open questions, that the ability of TE to estimate interaction delays, adding to the overlapping information provided by MIPAC, may favor the use of local TE in addition to or instead of MIPAC for intensive PAC analysis. However, in our opinion, there are a few technical considerations that first must be addressed before recommending local transfer entropy as a method for studying PAC in electrophysiological signals.

### 6.5. General Considerations for Approaching PAC Estimation Using Local Transfer Entropy

#### 6.5.1. Parameters Selection for Estimating Local TE

Perhaps the most controversial issue when computing transfer entropy is how to estimate its embedding parameters. As we did here, maximizing the AIS on each source to obtain the embedding parameters may potentially neglect multivariate effects between the past of both source and target signal. An alternative approach could be to optimize the AIS in the target signal and then find the source embedding that maximizes the TE from the source to the embedded target. In other words, one would find the source embedding that maximizes the conditional MI (CMI) between the source past and the next value in the target, conditioned on the target’s past. The CMI actually accounts for both redundancies between both past states (e.g., due to a common driver/input to both signals), as well as potential synergistic effects. Such an approach has, for example, been discussed recently by Novelli [62]. It remains here to investigate how this strategy may improve TE estimation in our PAC context. However, as we have already discussed, there is not yet any consensus on the best way to approach the embedding parameter estimation. In addition, note that the exhaustive search method used in this manuscript may not be practical to use when studying PAC in lengthy continuous signals. In such cases, methods like that proposed by Ragwitz [42] that estimate the dimension and delay of the embedding while minimizing the prediction error for future samples of the time series, may be the better option. This method is implemented in two of the most advanced, specialized, and widely used software tools for computing transfer entropy and other IT measures: JIDT [39] and Trentool [63].

Currently, the only way to accurately estimate local IT measures including transfer entropy is via the KSG estimator which also requires setting another parameter, the number of neighboring points *K* in the joint space spanned by the signal supports used to define the marginal neighborhoods to compute Equation (14). Unfortunately, there is currently no efficient approach to estimating this parameter, though once it is set properly (to avoid undersampling of points in the marginal spaces), the computed measure (either local TE or MI) is quite stable to the selection of [47]. To summarize the discussion of these parameters, we would like to stress the key importance of the selection and setting of both the embedding parameters and the number of neighbors K for accurate computation of local TE.

#### 6.5.2. Event-Related Data and TE

Here, we focused on characterizing the PAC process from the perspective of local TE applied to continuous signals. We did not consider the case of data segments time-locked to a set of similar stimulus events (i.e., event-related data epochs). We believe that these same methods could also be applied to such event-related data, estimation in this case taking advantage of the data geometry inherent in the matrix of similarly latency-aligned data windows (dimension, number of trials by latencies), as proposed by Gomez-Herrero [64] and applied by us for MIPAC estimation [3].

#### 6.5.3. The Importance of the Filtering Strategy

The two frequency components entering the PAC analysis are entirely determined by the filter bandwidths, which in turn depend on the filtering strategy used to extract them [61]. Ultimately, the selection of the bandwidth appears to be a critical part of preprocessing for TE computation. We have identified three ways in which the filtering strategy may negatively impact the PAC analysis outcome and, in particular, PAC measured by transfer entropy.

The first problem area is in selecting the bandwidth of the phase signal passband in order to satisfy the Hilbert transform narrowband requirement (ideally an almost sinusoidal activity). The choice of bandwidth for the phase component is constrained by the condition of the signal having a meaningful phase and is, therefore, often correctly chosen to be narrow [61]. However, the validity of the narrowband assumption for extracting high-frequency amplitude in most published PAC studies is questionable, as variability in high-frequency activity in brain signals very often does not have a narrowband characteristic [45,49]. Thus, using the Hilbert transform may yield non-meaningful amplitude estimations and thus potentially flawed PAC analysis [45,65,66]. On the other hand, shrinking the bandwidth to satisfy the Hilbert narrowband requirement has been shown to lead to misidentification by PAC of phase-frequency coupling—another form of cross-frequency coupling [67]. It has also been reported that narrowband filtering leads to an under-estimation of the information transfer delay [68]. In particular, Wollstadt reported a constant underestimation of the delay using narrowband filtered signals, a delay that increases the more narrow the band becomes (see Figure 7 in [69]). Thus, narrowband filtering may also be a potential source of error when reconstructing delays using TE. Although further investigation is needed, we hypothesize that narrowband filtering could be responsible for the latency bias shown in Figure 6. While much of the PAC literature uses the Hilbert transform to estimate both the phase and amplitude signals, other Fourier-based time-frequency methods might equally be applied to extract the amplitude signal of wideband high-frequency processes.

The second problem area is in selecting the bandwidth to capture the higher-frequency spectral features indicating an ongoing PAC process. Here, the bandwidth used to extract the high-frequency component should be wide enough to accommodate the sidebands peaks indicating modulation by lower-frequency activity. This requirement is key for successful PAC estimation and has led to strong PAC guidelines suggesting that, if a narrowband higher frequency component’s bandwidth does not include the sideband peaks produced by amplitude modulation by the lower frequency, then the presence of CFC cannot be detected [61]. Thus, as per Aru and colleagues, the solution to these requirements is to look for a sweet spot where the frequency dynamics shows a meaningful activity against small bandwidth settings [61].

Finally, methodological problems associated with isolation of frequency band activity by filtering have been largely discussed in the context of Granger Causality (GC) [70,71,72]. These issues may also affect TE computation, which for jointly Gaussian variables has been demonstrated to be equivalent to GC [73]. In particular, Weber [68] has shown that, when analyzing connectivity in a network setting, filtering and downsampling signals ahead of computing TE may reduce the number of detected connections. We believe that most of these caveats may be overcome by approaching TE findings with a hypothesis-driven analysis supported by stringent statistical testing, but further investigation is necessary in this regard. A recently proposed method has recommended addressing the problem of filtering in TE computation by implementing the filtering only when generating surrogate data representing the null-hypothesis of no information transfer at the frequencies of interest [74]. Studying how this approach will influence PAC analysis by local TE, where the signals entering the TE analysis are first bandpass filtered and Hilbert transformed (see Section Specific caveats in [74]), will be future work.

Here, despite the above-mentioned limitations, we have shown the potential of TE to address the study of delayed interactions in the PAC process. It should be noted that the same formulation used here for TE potentially allows studying PAC conditioned by other and/or more variables. This indeed may be a perfect approach to address the question of directionality between two processes when both may be driven by some third process. Although computing TE while naively conditioning on several other signals (e.g., instantaneous phase and amplitude values at other sources or channels) seems currently numerically infeasible [24], it is convenient to know that this may be an option in the future, given the ever-retreating compute horizon. In this regard, it is worth mentioning that recent proposals for the estimation of conditional and multivariate TE may ease the computational burden presented by the combinatorial explosions in the number of source combinations evaluated by implementing a non-uniform embedding strategy [62,75,76,77]. These strategies may represent a possible solution for the current problem in the TE-resolved PAC when conditioning by several instantaneous phase and amplitude values at other sources or channels.

## 7. Conclusions

Here, we used local transfer entropy (TE) to estimate and characterize phase-amplitude coupling in cortical local field activity. We used first simulated and then actual ECoG seizure data and, in both cases, found local TE peaks at onsets and offsets of PAC modulation periods estimated using MIPAC, our previously reported method for estimating time-resolved PAC. Although further investigation is needed, we hypothesize that information transfer indicated by TE may signal, or even facilitate, the coupling process. This mechanism should be the focus of further studies. We discussed some limitations we consider important to address before recommending that studies of PAC in electrophysiological signals rely on the use of local transfer entropy. Despite these cautions, we see real potential in the use of TE for the study of PAC, and specifically in the study of its interaction delays, which to date are an issue not widely discussed in the PAC literature. We are aware that, in our attempt to characterize PAC by features highlighted by local TE, we are leaving a number of open questions. We hope this initial investigation will help catalyze interest in the application of local TE to the brain PAC phenomenon, hopefully shedding light on the physiological role of PAC processes in the human brain.
